# Supplementary material for: Correlation driven near-flat band Stoner excitations in a Kagome magnet
Source: Nat Commun. 2022 Nov 28;13:7317. doi: 10.1038/s41467-022-34933-y (PMC9705307; doi:10.1038/s41467-022-34933-y)
Supplement: Supplementary file 1 — Supplementary Information [file 41467_2022_34933_MOESM1_ESM.pdf]

Supplementary Information for Correlation driven near-flat band Stoner excitations in a Kagome magnet

Abhishek Nag[1,*], Yiran Peng[2], Jiemin Li[1,3], S. Agrestini[1], H. C. Robarts[1,4], Mirian García-Fernández[1], A. C. Walters[1], Qi Wang[5], Qiangwei Yin[5], Hechang Lei[5], Zhiping Yin[2,†], and Ke-Jin Zhou[1,‡]

[1]Diamond Light Source, Harwell Campus, Didcot OX11 0DE, United Kingdom

[2]Department of Physics and Center for Advanced Quantum Studies, Beijing Normal University, Beijing 100875, China

[3]Beijing National Laboratory for Condensed Matter Physics and Institute of Physics, Chinese Academy of Sciences, Beijing 100190, China

[4]H. H. Wills Physics Laboratory, University of Bristol, Bristol BS8 1TL, United Kingdom

[5]Department of Physics and Beijing Key Laboratory of Opto-Electronic Functional Materials & Micro-Nano Devices, Renmin University of China, Beijing, China

[*]e-mail: abhishek.nag@diamond.ac.uk

[†]e-mail: yinzhiping@bnu.edu.cn

[‡]e-mail: kejin.zhou@diamond.ac.uk

S1 Sample information

$Co_3Sn_2S_2$ single crystal grown by the Sn flux method and used for the RIXS measurements is shown in Fig. S1a. The X-ray diffraction (XRD) peaks of the single crystal were measured using a Bruker D8 X-ray machine with Cu K_α radiation (λ = 0.15418 nm) at room temperature (Fig. S1b). All of the peaks can be indexed by the indices of (0, 0, *l*) lattice planes. It shows that the crystal surface is normal to the *c*-axis with the plate-shaped surface parallel to the *ab* plane. Fig. S1c shows the Laue diffraction pattern obtained from the single crystal at room temperature. Fig. S1d shows the Zero-Field-Cooled (ZFC) and Field-Cooled (FC) magnetisation data obtained as a variation of temperature under an applied magnetic field of 1 T along the *c*-axis. Ferromagnetic (FM) transition temperature T_C from magnetisation measurements was found to be 172 K.The inset shows magnetisation data obtained as a variation of magnetic field applied along the *c*-axis at 5 and 300 K.

S2 RIXS data fitting

RIXS data were fitted as described in Methods of main text. The RIXS line spectra and their fits are shown in panels a and b of Fig. S2, Fig. S3 and Fig. S4. The quasielastic peaks at zero energy transfer have been subtracted by fitting a Gaussian lineshape matching the experimental energy resolution, for better visualisation of the low energy peak S2. Due to the large damping factors of the peak S1 as shown in panel c of Fig. S2, Fig. S3 and Fig. S4, the associated undamped peak energies as shown in panel d of Fig. S2, Fig. S3 and Fig. S4, are higher than the peak maximum energies.

S3 High resolution RIXS data

Higher resolution ($\Delta E = 32$ meV) RIXS spectra were collected at Co L_3-edge to separate the quasielastic peak contribution from the low energy S2 peak as shown in Fig. S6, for both π (incident polarisation of X-ray parallel to the scattering plane) and σ (incident polarisation of X-ray perpendicular to the scattering plane). Both the quasielastic peak and S2 at ~ 0.04 eV show marginally higher intensity with the π polarisation for all (h, l) points.

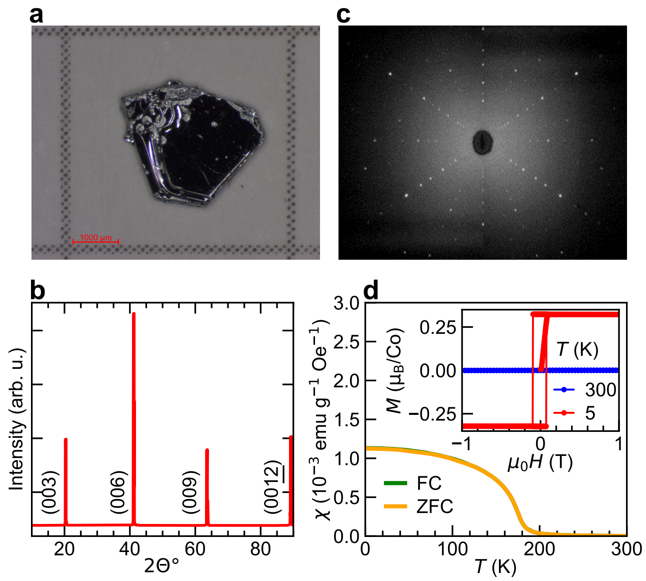

Figure S1. $Co_3Sn_2S_2$ sample characterisation. a, Photograph of a single crystal of $Co_3Sn_2S_2$. **b,** XRD peaks of the $Co_3Sn_2S_2$ single crystal. **c,** Laue diffraction pattern of the $Co_3Sn_2S_2$ single crystal. **d,** ZFC-FC data magnetisation data of $Co_3Sn_2S_2$ single crystal with magnetic field of 1 T applied along the *c*-axis. Inset shows magnetisation data obtained as a variation of magnetic field applied along the *c*-axis at 5 and 300 K.

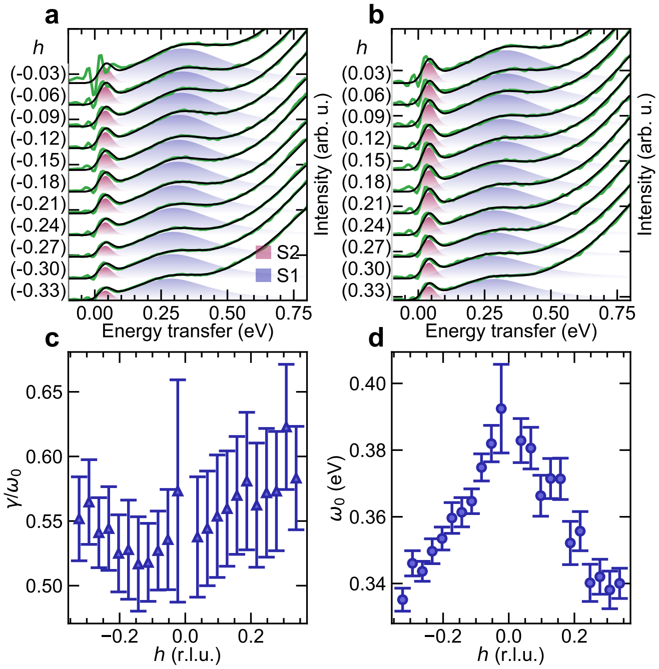

Figure S2. ***h*-dependent RIXS spectra of $Co_3Sn_2S_2$ at 23 K with fitting. a, b,** Low-energy RIXS spectra (green lines) along $\overline{\mathrm{M}}\,\overline{\Gamma}\,\overline{\mathrm{M}}$ direction for $h = -0.33$ to $h = 0.33$ at $l = 1.25$. The fitted quasielastic peak contributions have been subtracted. Black lines are aggregated least square fits of different low energy peak profiles and a high energy background. The shaded peak profiles S1 and S2 primarily represent Stoner excitations from spin-polarised flatbands. **c,** The damping factor and **d,** the undamped energy ω_0 of S1, extracted from fits of the RIXS spectra. Error bars are least-square-fit errors.

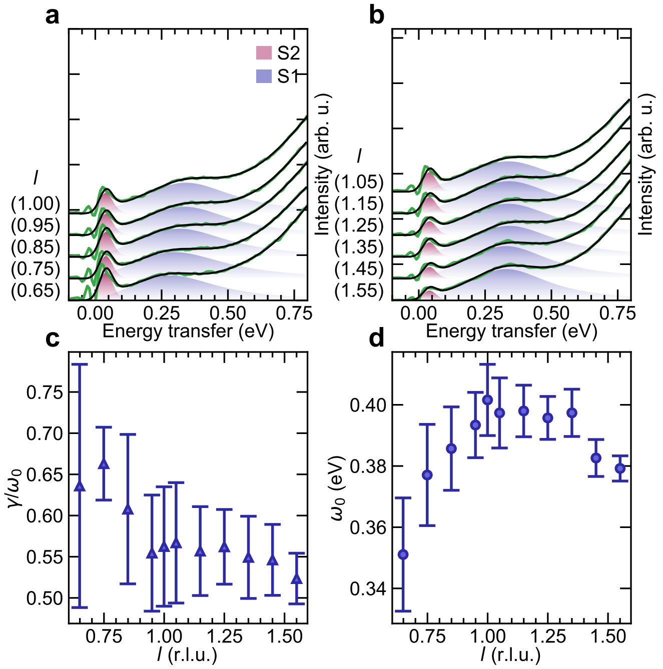

Figure S3. *l*-dependent RIXS spectra of $Co_3Sn_2S_2$ at 23 K with fitting. a, b, Low-energy RIXS spectra (green lines) along $\overline{\Gamma}\,\overline{A}$ direction for $l = 0.65$ to $l = 1.55$ at $h = 0.05$. The fitted quasielastic peak contributions have been subtracted. Black lines are aggregated least square fits of different low energy peak profiles and a high energy background. The shaded peak profiles S1 and S2 primarily represent Stoner excitations from spin-polarised flatbands. **c,** The damping factor and **d,** the undamped energy ω_0 of S1, extracted from fits of the RIXS spectra. Error bars are least-square-fit errors.

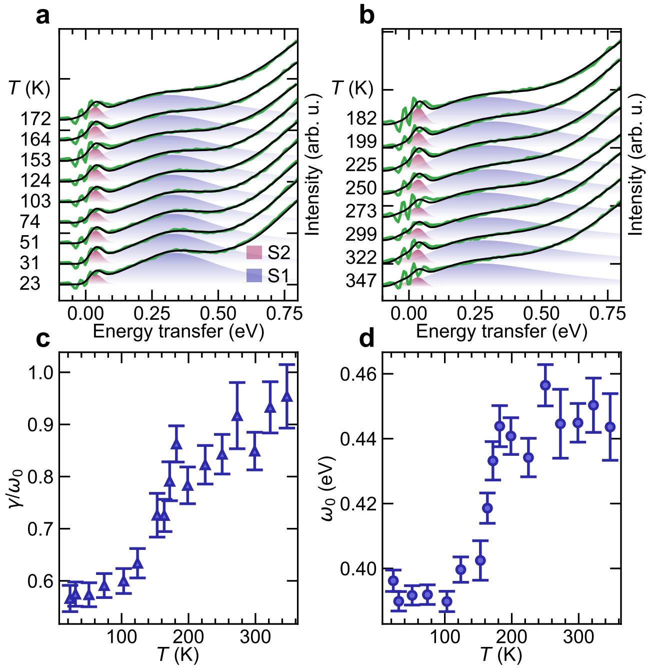

Figure S4. Temperature dependent RIXS spectra of $Co_3Sn_2S_2$ at $(0.025, 1.25)$ **with fitting. a, b,** Low-energy RIXS spectra (green lines) for different temperatures. The fitted quasielastic peak contributions have been subtracted. Black lines are aggregated least square fits of different low energy peak profiles and a high energy background. The shaded peak profiles S1 and S2 primarily represent Stoner excitations from spin-polarised flatbands. **c,** The damping factor and **d,** the undamped energy ω_0 of S1, extracted from fits of the RIXS spectra. Error bars are least-square-fit errors.

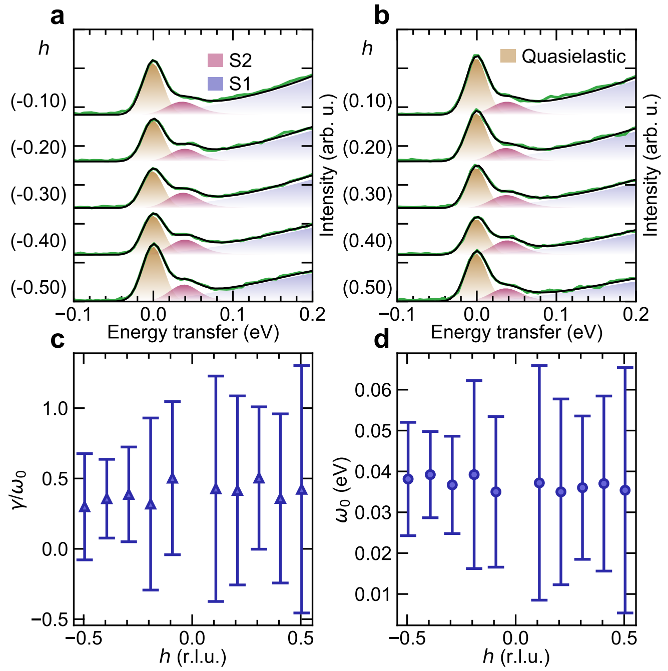

Figure S5. Low-energy high energy resolution RIXS spectra of $Co_3Sn_2S_2$ at 23 K with fitting. a, b, RIXS spectra highlighting the S2 peaks (green lines). Black lines are aggregated least square fits of different low energy peak profiles and a high energy background. The shaded peak profiles S1 and S2 primarily represent Stoner excitations from spin-polarised flatbands. **c,** The damping factor and **d,** the undamped energy ω_0 of S2, extracted from fits of the RIXS spectra. Error bars are least-square-fit errors.

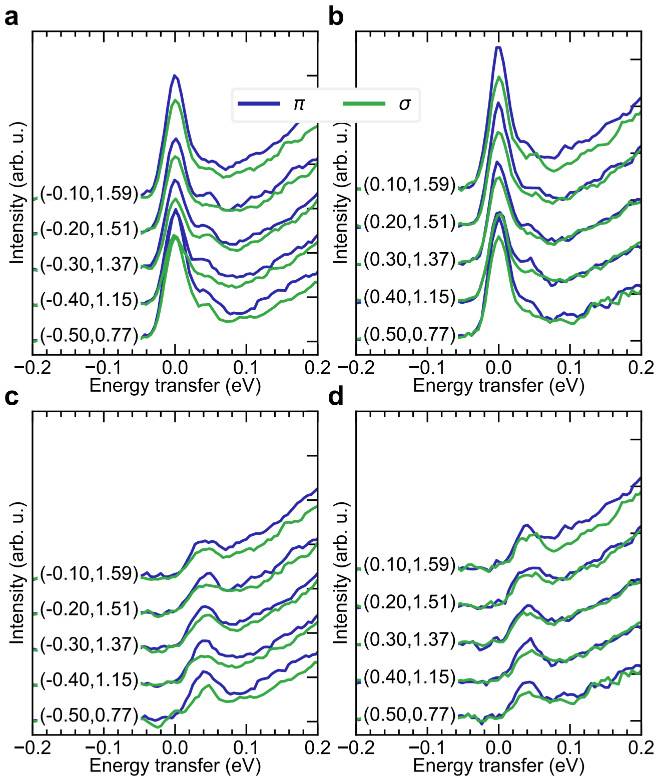

Figure S6. Polarisation dependent high energy resolution RIXS spectra of $Co_3Sn_2S_2$ at 23 K. a, b, High resolution ($\Delta E = 32$ meV) RIXS spectra on $Co_3Sn_2S_2$ collected with π and σ incident polarisations of X-ray. **c, d,** Same spectra with the fitted quasielastic peak contributions subtracted.

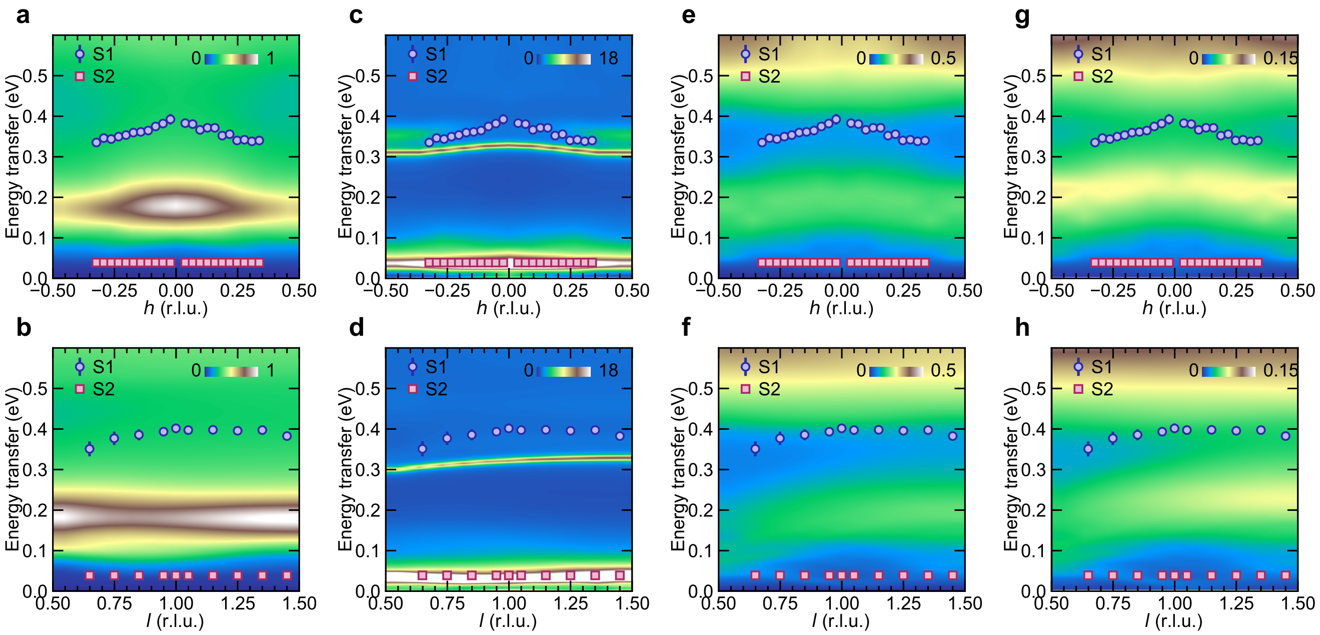

Figure S7. Calculated dynamic spin and charge susceptibilites in $Co_3Sn_2S_2$. Bare spin susceptibility intensity maps along the , $\bar{M}\,\bar{\Gamma}\,\bar{M}$ and **b,** $\bar{\Gamma}\,\bar{A}$ directions. Vertex corrected spin structure factor intensity maps along the **c,** $\bar{M}\,\bar{\Gamma}\,\bar{M}$ and **d,** $\bar{\Gamma}\,\bar{A}$ directions. Bare charge susceptibility intensity maps along the **e,** $\bar{M}\,\bar{\Gamma}\,\bar{M}$ and **f,** $\bar{\Gamma}\,\bar{A}$ directions. Vertex corrected charge susceptibility intensity maps along the **g,** $\bar{M}\,\bar{\Gamma}\,\bar{M}$ and **h,** $\bar{\Gamma}\,\bar{A}$ directions. The markers are the ω_0 values of S1 and S2 peaks extracted from least square fits of RIXS spectra. Error bars are least-square-fit errors.

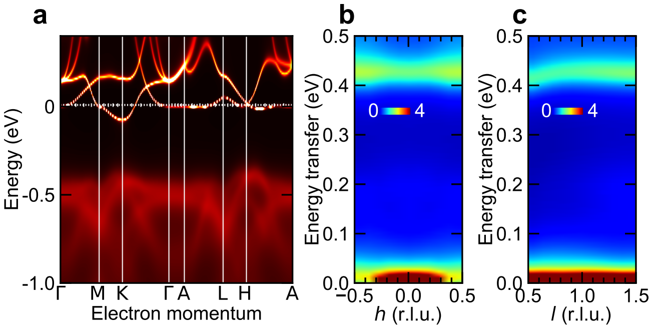

Figure S8. Paramagnetic state of $Co_3Sn_2S_2$. a, Calculated electronic bands in $Co_3Sn_2S_2$ in the paramagnetic state. Vertex corrected spin structure factor intensity maps along the **b,** $\bar{M}\,\bar{\Gamma}\,\bar{M}$ and **c,** $\bar{\Gamma}\,\bar{A}$ directions in the paramagnetic state.

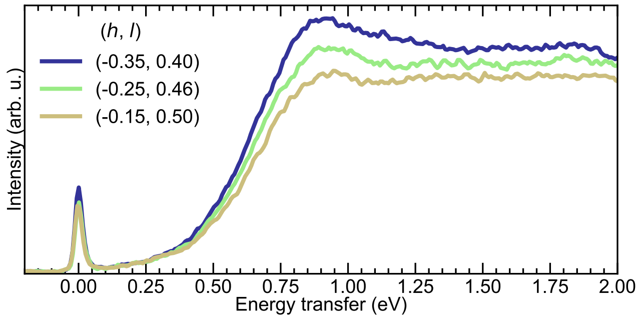

Figure S9. RIXS spectra of CoSn. Co L_3-edge RIXS spectra collected on paramagnetic flat band material CoSn at 20 K at different (h,l) points with σ polarisation.

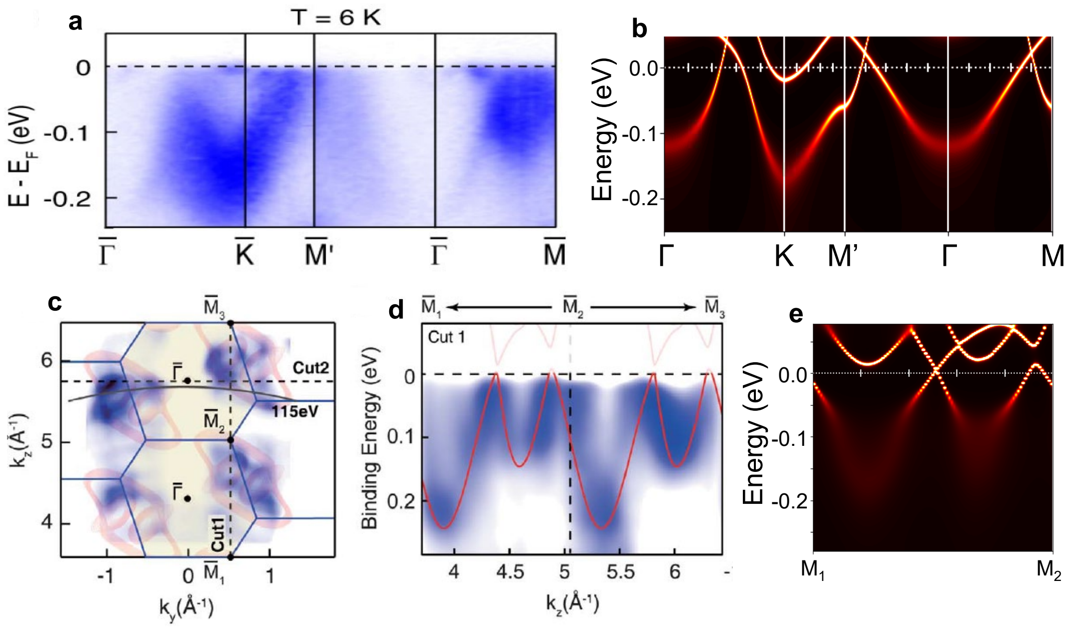

Figure S10. Comparison of ARPES data on $Co_3Sn_2S_2$ with band structure calculations. a, ARPES intensity plot along $\overline{\Gamma}\ \overline{K}\ \overline{M'}\ \overline{\Gamma}\ \overline{M}$, reprinted figure with permission from D. F. Liu *et al.*, Topological phase transition in a magnetic weyl semimetal, Phys. Rev. B, **104**, 205140 (2021). Copyright (2021) by the American Physical Society. **b,** Calculated electronic bands for the ferromagnetic state along Γ K M′ Γ M using DFT+DMFT in the present work. **c,** ARPES intensity plot along k_y-k_z plane with energy integration window from E_F-0.1 eV to E_F from from Liu *et al.* The black curve indicates the k_z momentum locations probed by 115-eV photons. The dashed line marked as 'cut1' indicates the momentum direction $\overline{M}_1\ \overline{M}_2\ \overline{M}_3$ of the ARPES data shown in panel **d** from D. F. Liu *et al.*, Magnetic Weyl semimetal phase in a Kagomé crystal, Science, **365**, 1282-1285 (2019). Reprinted with permission from AAAS. The red curves in panel **d** are from DFT calculations, where the calculated bandwidth was renormalised by a factor of 1.43 and the energy position was shifted to match the experiment. **e** Calculated electronic bands for the ferromagnetic state along M_1 M_2 using DFT+DMFT in the present work.
